# Distinct ERP signatures for singular “they” and gender violations

**DOI:** 10.3389/fpsyg.2026.1797415

**Published:** 2026-04-30

**Authors:** Joanna Morris, Jesse Snedeker, Grusha Prasad

**Affiliations:** 1 School of Cognitive Science, Hampshire College, Amherst, MA, United States; 2 Department of Psychology, Providence College, Providence, RI, United States; 3 Department of Psychology, Harvard University, Cambridge, MA, United States; 4 Department of Cognitive Science, Johns Hopkins University, Baltimore, MD, United States; 5 Department of Computer Science, Colgate University, Hamilton, New York, NY, United States

**Keywords:** event-related potentials (ERPs), gender agreement, language change, P600, pronoun processing, referentiality, singular they

## Abstract

**Introduction:**

The English pronoun system is undergoing rapid change, with singular “*they”* increasingly used to refer to specific individuals. A key question is how this innovative usage is processed online, particularly when paired with gendered antecedents. The critical issue is whether singular “*they”* incurs processing costs similar to canonical gender mismatches or instead engages a distinct interpretive profile that avoids the neural signatures typically elicited by incongruent uses of “he” and “she”.

**Methods:**

We addressed this question using event-related potentials (ERPs) in readers with high familiarity with singular “*they”*. Participants listened to sentences containing reflexive pronouns whose antecedents varied in gender specificity (gendered vs. non-gendered) and referential status (referential vs. bound-variable).

**Results:**

Across both the P600 (500–800 ms) and N400/Nref (300–500 ms) time windows, gender-incongruent reflexives elicited robust neural signatures of processing difficulty that depended on antecedent type. Referential mismatches with proper names (e.g., “John…herself”) produced frontal negativities consistent with referential integration difficulty, whereas bound-variable mismatches with quantified antecedents (e.g., “every woman…himself”) elicited large P600 effects characteristic of morphosyntactic repair. In contrast, singular “themselves” patterned differently from these gender mismatch responses in gendered antecedent contexts. In referential contexts, the N400/Nref response to singular “themselves” was intermediate between those for gender-congruent and gender-incongruent forms, consistent with a gradient of predictability. In bound-variable contexts, singular “themselves” patterned closely with gender-congruent reflexives, showing no evidence of a violation response.

**Discussion:**

These findings suggest that, for speakers with substantial exposure, singular “themselves” does not behave like a canonical agreement mismatch, even in contexts that strongly penalize gender-incongruent forms. This pattern counters the intuition that gender-neutral reflexives necessarily impose a processing cost when paired with gendered antecedents.

## 1 Introduction

The use of third-person pronouns in English is undergoing rapid change, driven in part by increasing awareness of gender diversity and a growing need for inclusive language. Traditionally, English has lacked a gender-neutral singular pronoun for referring to specific, animate individuals, so many speakers have turned to “*they”* to fill this gap. However, earlier research has shown that mismatches in grammatical features–such as gender and number–between pronouns and their antecedents often lead to processing difficulty, particularly in real-time language comprehension. This has raised concerns that singular uses of “*they*”, especially when referring to definite or gendered individuals, might engage similar mechanisms associated with feature conflict, reanalysis, or referential difficulty. At the same time, language allows for considerable flexibility, and growing exposure to singular “*they*” may reduce or qualitatively alter how such configurations are processed. An open question, therefore, is not simply whether singular “*they*” incurs processing costs, but whether it patterns like canonical gender mismatches or instead engages a distinct interpretive profile that avoids the neural signatures typically elicited by incongruent uses of “*he*” and “*she*”. While existing studies have begun to address this issue, most rely on offline judgment tasks (e.g., Arnold et al., 2021), leaving open the question of how singular “*they*” is integrated during online comprehension. Event-related potentials (ERPs) offer a well-established method for indexing real-time sensitivity to morphosyntactic and referential relationships during language processing. Moreover, we now have access to populations with frequent exposure to both gender non-conforming individuals and to the singular use of “*they*”, providing a unique opportunity to investigate how familiarity shapes neural responses to pronoun-antecedent relations.

In the following sections of the Introduction, we begin by reviewing ERP research on pronoun processing, focusing on the components most relevant to gender and number agreement. Next, we consider current research on how English speakers interpret singular “*they*”, highlighting distinctions between referential and bound-variable uses (e.g., pronouns referring to specific individuals vs. those bound by quantificational antecedents) and their consequences for comprehension. We then turn to how socio-pragmatic factors—including gender stereotypes and shifts in language norms—interact with grammatical processing. Finally, we outline how the current study addresses unresolved questions by integrating these strands of research.

### 1.1 ERP evidence on pronoun processing

ERP research provides insight into how the brain processes pronoun-antecedent mismatches. Two components are particularly relevant to the present study, but with distinct theoretical roles: the P600 as the canonical response to categorical agreement violations, and the N400/Nref as an alternative signature associated with referential ambiguity or integration difficulty.

The P600—a late, broadly distributed positivity peaking between roughly 500–800 ms—emerges reliably in response to clear gender or number violations across languages and is generally interpreted as reflecting syntactic reanalysis or morphosyntactic repair (Osterhout and Mobley, 1995; Osterhout et al., 1997; Pablos et al., 2015; Lamers et al., 2008). Its amplitude can be modulated by task demands, with larger effects when participants make explicit grammaticality judgments. In contrast, negative-going components in the 300-500 ms window are sensitive to a very different set of variables. The N400, a centro-parietal negativity peaking around 400 ms, is classically associated with semantic integration difficulty but has more recently been linked to the updating that occurs when lexical information is unpredictable (Kutas and Federmeier, 2011; Nour Eddine et al., 2024). The Nref is a sustained frontal negativity, beginning around the same time as the N400, which is associated with difficulty linking a pronoun to a specific antecedent (Van Berkum et al., 2003).

While the N400 and Nref differ in their canonical descriptions, the N400 being centro-parietal and the Nref more sustained and frontal, many researchers have noted that the two constructs cannot be cleanly disentangled in practice. Both (Nieuwland 2014) and (Filik et al. 2008) observed negativities with a mix of the topographic and functional features associated with these two components. For this reason, in the present study we refer to negative-going components in the classic N400 window as N400/Nref effects, making no commitment as to which effect is observed, whether these effects are distinct, or whether they are part of a wider family of N400 effects that reflect similar processes at different levels of representation (see Kutas and Federmeier, 2011, for discussion). Where the distinction between these two components is theoretically relevant to the interpretation of specific effects, we note it in the text.

A consistent finding across languages is that the P600 is the dominant ERP correlate of pronoun-antecedent gender or number mismatches, and thus the primary neural signature predicted when mismatches are interpreted as violations of agreement. In English, (Osterhout and Mobley 1995) demonstrated that sentences such as “*The aunt heard that he…*” elicited robust P600s when participants judged them unacceptable, an effect interpreted as syntactic reanalysis at the pronoun. Comparable late positivities have been reported in Spanish for both gender and number mismatches, with gender violations often producing larger amplitudes than number violations (Barber and Carreiras, 2005), and in Mandarin Chinese, where gender mismatches yield an earlier and larger P600 than number mismatches (Xu et al., 2013).

However, not all mismatches are treated as violations. For example, in a sentence like “*The aunt heard that he…*”, the pronoun he can be interpreted in two distinct ways. On one reading, it introduces a new referent—someone other than “*the aunt*”, whose identity the listener may or may not be able to infer from context. On another reading, the pronoun is understood as attempting to refer back to “*the aunt*”—a coreference interpretation—in which case it constitutes a feature mismatch (misgendering or misnumbering), since “*he*” does not agree with the features of the antecedent just provided. When a mismatch is not interpreted as a categorical agreement violation but instead as referential ambiguity or failed semantic integration, the ERP response often shifts away from a P600 toward an N400/Nref, particularly in languages with less morphologically rich gender marking. For example, in English, (Nieuwland 2014) found that under comprehension-focused reading conditions, without trial-by-trial acceptability judgments, gender incongruities (e.g. “*The boy thought that she…*”) frequently elicited frontal negativities associated with referential ambiguity, whereas P600s appeared mainly in low-working-memory readers who persisted with a coreference interpretation despite the mismatch.

When reflexive pronouns are used, the possibility of introducing a new antecedent is eliminated, such that any gender mismatch at the reflexive unambiguously challenges the established coreference relation. Under these circumstances, gender mismatches result in P600 effects both for definitional mismatches —where the antecedent 's gender is morphologically or biologically fixed (e.g., “*actress…himself* ”)— and for stereotypical mismatches, where gender is implied by social norms or occupational bias (e.g., “*architect…herself* ”). Stereotypical mismatches have also been reported to elicit an N400/Nref — a pattern we return to in the General Discussion (Canal et al., 2015).

Finally, stereotype-based effects are further modulated by individual differences. (Canal et al. 2015) reported that variability in ERP responses to stereotype violations covaried with self-reported gender-related traits, as measured by the Bem Sex Role Inventory (Bem, 1974). Specifically, participants scoring higher on the expressiveness (“femininity”) dimension—a scale indexing endorsement of stereotypically expressive or communal traits—tended to show more positive-going responses in the P600 time window, whereas those scoring lower showed more pronounced anterior negativities. Importantly, these effects emerged as higher-order interactions and were interpreted by the authors as reflecting differences in interpretive strategy, rather than a direct mapping between personality traits and specific ERP components. The findings nevertheless underscore that social attitudes and self-construal can bias whether a stereotype violation is processed as a failure of agreement or as a referential challenge.

Together, this literature suggests that while the P600 constitutes the canonical neural response to pronoun-antecedent agreement violations, the presence of an N400/Nref can serve as a diagnostic marker that comprehenders have instead engaged in referential integration or ambiguity resolution.

### 1.2 Singular “*they*” processing

Recent psycholinguistic and theoretical work has deepened our understanding of how English speakers process the singular use of “*they*”. A central distinction in this literature is between referential and bound-variable uses, which differ in meaning and in processing profile. In referential contexts, “*they*” refers to a specific individual. For example, in the sentence “*Alex promised themselves that they would try harder next time*”, the pronouns “*they*” and “*themselves*” refer to Alex. In bound-variable contexts, such as “*Every runner thought they were the fastest*”, “*they*” refers generically to each member of a group introduced by a quantifier, rather than to any one specific individual. This distinction has a long-standing history in English. Bound-variable uses of singular “*they*” with gendered antecedents have been attested for centuries, as in Shakespeare's line: “*There's not a man I meet but doth salute me / As if I were their well-acquainted friend (Shakespeare, 3 ed)*”. Referential singular “*they*”, by contrast, is less well-attested historically and appears to reflect a more recent grammatical and social development (see, Bjorkman, 2017).

Psycholinguistic studies converge on a consistent pattern: bound-variable singular “*they*” is processed with relative ease, even when the antecedent is gendered. In acceptability rating studies, sentences like “*Every policeman thought that they were the winner*” are rated as acceptable as gender-matched forms like “*Every policeman thought that he was the winner*”. Self-paced reading experiments show no processing slowdown in such contexts (Han and Moulton, 2022; Moulton et al., 2022). In contrast, referential singular “*they*” often does incur processing costs, particularly when the antecedent is strongly gendered. Self-paced reading and eye-tracking studies find slower processing when “*they*” refers to an individual whose gender is either known or presupposed (Foertsch and Gernsbacher, 1997). These effects are especially pronounced when the antecedent is definitionally gendered (e.g., “*the man*”, “*the sister*”), although stereotypically gendered nouns (e.g., “*the nurse*”, “*the mechanic*”) can also trigger disruptions (Canal et al., 2015; Kreiner et al., 2008; Reali et al., 2015). This suggests that lexically encoded gender exerts a stronger influence on processing than culturally stereotyped gender, though both are relevant.

These differences are often explained in terms of the featural representation of gender in pronouns and antecedents (Bjorkman, 2017; Ackerman, 2019). Singular “*they*” is underspecified for gender, whereas “*he*” and “*she*” are specified as masculine and feminine, respectively (Bjorkman, 2017). In bound-variable constructions, pronouns correspond to reduced structural representations that may lack gender features, allowing “*they*” to avoid feature conflict. In referential contexts, by contrast, pronouns correspond to fully specified determiner phrase (DP) representations that include gender features, creating a potential mismatch when “*they*” is paired with a gendered antecedent. At the level of interpretation, such mismatches can hinder integration with an established discourse referent, contributing to reduced acceptability and increased processing cost (Foertsch and Gernsbacher, 1997; Nieuwland, 2014). These contrasts between bound-variable and referential uses underscore the importance of antecedent type, since some antecedents create stronger expectations for gender congruence than others.

A further consideration concerns the asymmetry between masculine and feminine pronouns in contexts where gender is not specified by the antecedent. In English, the masculine form has historically functioned as the default or unmarked option for generic and bound-variable reference—as in the prescriptive generic “*he*”—whereas the feminine form carries stronger gender-marking and is more restricted in distribution (Bjorkman, 2017). Although prescriptive generic “*he*” has declined substantially in contemporary usage, with singular “*they*” increasingly filling the generic default role (Foley and Ahn, 2024), the distributional asymmetry between masculine and feminine reflexives in non-gendered contexts remains robust in corpus data (Davies, 2008). From a markedness perspective, this asymmetry predicts that feminine-marked reflexives should impose a stronger processing cost than masculine-marked reflexives in bound-variable and generic contexts, since the introduction of a marked feminine feature in a context that does not license gender specification represents a stronger interpretive commitment than the introduction of the historically default masculine. This prediction applies most clearly to non-gendered antecedent conditions (e.g., “*everyone*”), where the antecedent itself provides no gender cues; its application to gendered antecedent conditions (e.g., “*every woman*”) is less straightforward, since in those contexts the reflexive either matches or mismatches an antecedent that already specifies gender. We return to the asymmetric behavior of feminine reflexives in the Results and Discussion.

Most research on the gender-neutral use of “*they*” has relied on behavioral methods; to the best of our knowledge, there are no ERP studies directly examining the gender-neutral use of “*they*” or “*themselves*”. The closest relevant work is by (Filik et al. 2008), who compared responses to “*they*”, “*he*”, and “*she*” when these pronouns were introduced without a prior antecedent (e.g., “*The in-flight meal I got was more impressive than usual. In fact, she/they courteously presented the food as well*”). They found that gendered pronouns (“*he/she*”) elicited a late fronto-central positivity (FP600) around 750 ms, which the authors linked to the additional processing required to introduce a new, inferred referent into the discourse representation. “*They*” in the same no-antecedent condition did not elicit this late positivity, suggesting that readers can readily link “*they*” to a general or inferred group–such as “*the airline*” or “*the staff* ”—without needing to identify a specific individual.

The same study also included a control condition in which a prior referent was present (“*The in-flight meal I got from the stewardess was more impressive than usual. In fact, she/they courteously presented the food as well*”). This control condition bears directly on questions raised by work on referential ambiguity and coreference commitment. If “*they*” were initially interpreted as requiring a plural referent, one might have expected a discourse-related positivity for “*they*” relative to “*she*” in this context, reflecting the need to revise or update the discourse representation. No such positivity was reported. However, the absence of this effect is difficult to interpret, since non-reflexive “*they*” may still be understood as maintaining referential underspecification, even when a singular antecedent is available.

Crucially, whether or not an antecedent was provided, “*he/she*” elicited a larger N400-like negativity than “*they*”. (Filik et al. 2008) interpret this effect as reflecting a stronger commitment to identifying a specific discourse referent for gendered singular pronouns, resulting in greater integration difficulty when such commitment is not immediately supported. By contrast, “*they*” permits a looser interpretive strategy that tolerates referential ambiguity. In this respect, the findings parallel those of Nieuwland (2014): when readers do not persist in a coreference interpretation, processing difficulty is expressed as an N400/Nref rather than a later positivity associated with reanalysis or discourse updating. Together, these results suggest that “*they*” biases comprehenders toward an ambiguity-tolerant interpretive stance, thereby avoiding both early integration costs and later revision processes.

### 1.3 Social and experiential influences on pronoun processing

While ERP studies reveal how grammatical and referential features shape pronoun processing, they also point to an important role for socio-pragmatic factors. Readers' expectations about gender, shaped by stereotypes, social norms, and individual experience, can influence interpretation even in the absence of formal mismatches. Sentences like “*The nurse prepared himself for the procedure*” or “*The mechanic found herself confused*”, though grammatically well-formed, often elicit P600-like positivities, reflecting reanalysis processes triggered by stereotype violations (Osterhout et al., 1997). These stereotype-related ERP effects vary across individuals, potentially shaped by age, cultural background, gender identity, and beliefs about gender roles. (Osterhout et al. 1997) found that female participants showed larger P600 effects to gender mismatches than did male participants, while (Canal et al. 2015) reported that younger adults and individuals with more progressive gender attitudes tended to show attenuated P600 responses to stereotype violations. Although the underlying mechanisms remain unclear, these findings suggest that social experience and attitudinal factors can modulate whether stereotype incongruities trigger reanalysis processes during comprehension.

Pragmatic context also matters: (Arnold et al. 2021) examined the effect of explicit pronoun introduction (e.g., “*This is Alex, and “they” use they/them pronouns*”) on acceptability judgments and singular construal of “*they*”. Explicit introduction increased the likelihood that “*they*” was interpreted as singular. By contrast, neither repeated exposure within the experiment nor self-reported prior familiarity with nonbinary individuals reliably influenced interpretation when pronoun cues were implicit.

(Konnelly and Cowper 2020) propose a staged trajectory in the acceptability of referential singular “*they*” with gendered antecedents. In an initial stage, speakers accept singular “*they*” only when the referent's gender is unknown or irrelevant, while in later stages its use extends to clearly gendered referents. This progression appears to reflect broader social and demographic change, with younger speakers, as well as speakers who identify as nonbinary or transgender, especially likely to judge singular referential “*they*” as fully acceptable in contexts that might challenge more conservative grammatical intuitions. These trajectories also highlight that antecedent type, particularly the degree to which gender is lexically specified, plays a critical role in pronoun resolution.

(Bjorkman 2017) observes that while innovative use of singular “*they*” extends to antecedents that are singular, definite, and specific, many speakers who accept “*they*” in these contexts nonetheless resist its use with proper names or strongly gendered nouns. This restriction reflects the special status of proper names in Bjorkman's account of singular “*they*” processing. Both (Bjorkman 2017) and (Konnelly and Cowper 2020) argue that proper names introduce highly specific, uniquely identifying discourse referents, and that stereotypically gendered names carry stable gender cues. As a result, proper names may function as privileged antecedents, such that pronoun interpretation mechanisms draw heavily on the features they provide to anchor reference in discourse (Cunnings et al., 2015). Consistent with this view, studies of reflexive pronoun resolution in Mandarin report early and reliable mismatch effects for name antecedents, suggesting heightened sensitivity to feature conflict when reference is anchored to a named individual (Su et al., 2016). In Bjorkman's account, these strong feature-based expectations render the use of singular “*they*” with proper-name antecedents especially difficult, even for speakers who otherwise accept they with definite and specific common nouns.

Together, these findings point to a system in which pronoun interpretation is dynamically shaped by the interaction of grammatical features, pragmatic context, and sociolinguistic experience. However, much of the existing evidence comes from offline acceptability judgments or behavioral measures, leaving open the question of how these social and experiential factors influence real-time neural processing. In particular, it remains unclear how singular uses of “*they*” are integrated online, and whether they elicit neural responses associated with feature conflict, reanalysis, or discourse updating despite their widespread use.

### 1.4 The current study

In this study, our goal is to determine how singular “*they*” is processed in real time when used with singular antecedents—particularly those that are strongly gendered—in a cohort of participants with considerable exposure to gender-diverse individuals and inclusive language practices. Using event-related potentials (ERPs) as our dependent measure, we examined whether singular “*they*” engages the same neural mechanisms that are reliably observed for canonical pronoun-antecedent feature mismatches, and whether its processing profile differs across referential and bound-variable contexts.

By combining reflexive pronouns, which require coreference, with both gendered and non-gendered antecedents in referential and bound-variable constructions, we were able to directly compare the neural responses elicited by gender-congruent pronouns, gender-incongruent pronouns, and singular “*they*”. This design allowed us to assess whether singular “*they*” patterns like canonical agreement mismatches, like congruent forms, or instead exhibits a distinct processing profile.

Based on prior ERP research on pronoun resolution and agreement, we formulated the following predictions. First, gender-incongruent pronouns (“*he*”/“*she*”) were expected to elicit robust mismatch responses relative to gender-congruent pronouns, expressed canonically as a P600 in bound-variable contexts and as an N400/Nref in referential contexts. Second, if singular “*they*” avoids canonical feature-mismatch processing, its neural profile was expected to differ from that of gender-incongruent pronouns across both antecedent types. Third, we did not have a strong prediction as to whether singular “*they*” would pattern identically to gender-congruent pronouns; rather, we anticipated that any differences between these forms would be smaller than the contrast between singular “*they*” and clearly incongruent pronouns. Finally, we expected antecedent type to modulate these effects, reflecting differences in how reference is established for specific individuals versus quantified expressions. We further anticipated that effects in the non-gendered antecedent conditions would not be symmetric across pronoun types. Based on the markedness asymmetry discussed in Section 1.2, feminine-marked reflexives were expected to show stronger mismatch-related responses than masculine-marked reflexives in bound-variable contexts, where the introduction of marked gender features is least licensed by the antecedent.

#### Design provenance and analytic scope

The present experiment was originally designed to test predictions about late, violation-related ERP responses–specifically the P600—given its well-established association with categorical gender agreement mismatches in pronoun processing. Accordingly, analyses of the 500-800 ms window were specified a priori and treated as the primary, confirmatory tests of our hypotheses. During inspection of the grand-average waveforms and scalp distributions, however, we observed systematic anterior negativities in some conditions, particularly in referential contexts. Given extensive prior evidence linking such negativities to referential ambiguity and integration difficulty, we conducted complementary analyses in the 300–500 ms N400/Nref time window to characterize the *type* of processing difficulty associated with different pronoun-antecedent relations. These analyses were not part of the original analysis plan and are therefore interpreted descriptively, as diagnostic of whether effects reflect violation-driven reanalysis (P600) or earlier referential integration processes (N400/Nref), rather than as independent tests of equivalence between pronoun forms.

## 2 Method

### 2.1 Participants

We surveyed 256 volunteers from Hampshire College and the Five Colleges Consortium community regarding their gender identity, knowledge of gender-neutral pronouns, and the frequency with which they interacted with individuals who identify as nonbinary. Each of these factors was assigned a numerical score, and scores were summed to yield a composite measure of participants' familiarity with nonbinary individuals, with higher scores indicating greater familiarity. Possible scores ranged from 3 to 13. From this pool, we recruited 45 participants with familiarity scores of 10 or higher corresponding to the upper range of the score distribution and reflecting consistently high familiarity across survey dimensions. All participants were right-handed, monolingual native speakers of English. Twenty-three participants identified their gender as nonbinary; the remaining 22 identified as male (*n* = 15) or female (*n* = 7). The two groups did not differ in linguistic proficiency or linguistic background. Data were collected across two sessions of approximately three hours each, and participants received $50 in total for completing both sessions. All materials and procedures were reviewed and approved by the Institutional Review Board at Hampshire College.

### 2.2 Materials

For gendered items, antecedents were either referential (e.g., “*Mary*” …) or bound-variable (e.g., “*Some woman*” …), and were paired with reflexive pronouns that were gender-congruent (“*herself* ”), gender-incongruent (“*himself* ”), or gender-neutral (“*themselves*”); “*themselves*” was used as the gender-neutral reflexive form throughout, rather than the alternative singular form “*themself* ” (see Section 4.3 for discussion). For non-gendered items, antecedents were likewise referential (e.g., “*The runner*” …) or bound-variable (e.g., “*Someone*” …), but lacked inherent gender specification and were paired with masculine (“*himself* ”), feminine (“*herself* ”), or gender-neutral (“*themselves*”) reflexives. Each item set followed a 2 (antecedent type) × 3 (reflexive type) design, with 50 sentence frames per condition (300 sentences per set; see Table 1), yielding 600 experimental sentences in total. All stimuli and data are publicly available on OSF at https://osf.io/2vjyp/.

**Table 1:** Examples of stimuli by antecedent type and referential status.

Antecedent Type	Gender-congruent / masculine-marked	Gender-incongruent / Feminine-marked	Gender-neutral (*themselves*)
Referential gendered	John / Mary decided to treat himself / herself to sushi.	John / Mary decided to treat ^*^herself / ^*^himself to sushi.	John / Mary decided to treat themselves to sushi.
Bound-variable gendered	Every man / woman must learn to stand up for himself / herself.	Every man / woman must learn to stand up for ^*^herself / ^*^himself.	Every man / woman must learn to stand up for themselves.
Referential non-gendered	The stranger poured himself a cup of coffee.	The stranger poured herself a cup of coffee.	The stranger poured themselves a cup of coffee.
Bound-variable non-gendered	Someone in the group needs to pull himself together.	Someone in the group needs to pull herself together.	Someone in the group needs to pull themselves together.

Columns represent the three reflexive pronoun conditions: gender-congruent, gender-incongruent (for gendered antecedents only), and gender-neutral. Slashes indicate alternative forms; starred forms indicate gender mismatch.

In addition to these 600 experimental sentences, participants heard 200 sentences using the same sentence frames with lexically different antecedents of the same structural type, 800 sentences from a separate set of idiomatic stimuli tested in the same sessions but not analysed in the present paper, and 400 neutral filler sentences without pronouns or reflexives. Filler sentences were repeated twice, resulting in a total of 2,400 sentences per participant. Stimuli were presented across two sessions of 1,200 sentences each (600 experimental), spaced one to two weeks apart. Each session was divided into 10 blocks of 120 sentences. Items were pseudo-randomly assigned to blocks and sessions to balance antecedent types; for each sentence frame, all antecedent-reflexive versions appeared in the same session but never within the same block. Sentence order within blocks was randomized separately for each participant.

To minimize excessive blink related artifacts in the EEG recording, all sentences were presented auditorily, using OpenSesame (Mathôt et al., 2012) and Expyriment (Krause and Lindemann, 2014)^1^. The sentences were recorded by a native speaker of American English over several sessions. The recordings were normalized to have the same volume level and split into separate files at the onset of the /s/ phoneme in the reflexive pronoun (the point at which the reflexive pronoun becomes uniquely identifiable) using the Praat phonetics software package (Boersma and Weenink, 2001). The EEG signal was time locked to this point.

### 2.3 Procedure

#### 2.3.1 Experimental procedure

Participants sat at a comfortable distance from the computer screen and listened to the sentences through headphones. To ensure that participants were attending to the stimuli, 120 trials in each session were followed by a “*yes/no*” comprehension question, with correct responses equally distributed between “*yes*” and “*no*”. Participants responded by pressing one of two buttons on a gamepad controller. The buttons corresponding to “*yes*” and “*no*” responses were switched across sessions, with order counterbalanced across participants. Comprehension questions were presented in the center of the screen in a white font on a black background. To minimize eye movements during each trial, participants were instructed to keep their eyes focused on a spot in the center of the screen marked by a triangular sticker.

Each sentence was followed by a 500-ms inter-trial interval (ITI), unless the reflexive pronoun occurred as the final word of the sentence, in which case the ITI was extended to 1500 ms. On trials with comprehension questions, the question was presented after a 1500-ms ITI, and participants were given 1,000 ms to respond. To minimize eye-blink artifacts, participants were instructed to refrain from blinking during trials and were given an additional 500-ms “blink” break after every third trial, signaled by the characters (- -) presented on the screen. This was followed by another 500-ms break. Participants were given the opportunity to take an extended, self-timed break after every block.

#### 2.3.2 ERP data acquisition and pre-processing procedure

EEG activity was recorded from 30 scalp locations using tin electrodes mounted in an elastic cap (Electro-Cap International). In addition to these scalp electrodes, five supplementary electrodes were used: two placed on the left and right mastoids, two on the outer canthus of the left and right eye, and one beneath the left eye. During recording, signals were referenced online to the right mastoid and subsequently re-referenced offline to the average of both mastoids. Horizontal eye movements were monitored using the canthus electrodes, while vertical eye movements and blinks were monitored via the electrode beneath the left eye. Data were sampled at 1,000 Hz and downsampled to 200 Hz during preprocessing. Online filtering was applied between 0.1 and 100 Hz, and an additional offline bandpass filter of 0.1–30 Hz (−3 dB) was used. A 200-ms pre-stimulus baseline was applied to reduce temporal drift. Epochs were 1,000 ms in duration; however, artifacts such as blinks or excessive muscle activity were rejected only if they occurred within the first 800 ms following stimulus onset, corresponding to the time window of interest. Participants with more than 35% of trials rejected across sessions were excluded from further analysis (three participants in total). Because of experimenter error, several sentences were repeated (no more than five per condition); in these cases, the second presentation was excluded from averaging. Following artifact rejection, ERPs were averaged within condition for each participant, and mean amplitudes were extracted between 500 and 800 ms post-stimulus onset for the primary P600 analysis and between 300 and 500 ms for the exploratory N400/Nref analysis (see Section 3.1). Analyses of the P600 component were based on a centro-parietal electrode cluster comprising TP7, CP3, CPz, CP4, TP8, P7, P3, Pz, P4, and P8. Analyses of the N400/Nref component were based on a fronto-central electrode cluster comprising F7, F3, Fz, F4, F8, FT7, FC3, FCz, FC4, and FT8. All preprocessing was carried out using EEGLAB (Delorme and Makeig, 2004) and ERPLAB (Lopez-Calderon and Luck, 2014).

### 2.4 Statistical analysis

The experiment was analyzed in two separate models–one for gendered items and one for non-gendered items–to avoid an overly complex design with 12 conditions and to facilitate clearer interpretation within each antecedent type. Statistical analyses were conducted using linear mixed-effects modeling in R version 4.4.2 (R Core Team, 2024) within RStudio (Team, 2024), employing the afex package (Singmann et al., 2024). A mixed-effects approach was chosen over traditional repeated-measures ANOVA because it accommodates unbalanced data, accounts for subject-level variability, and does not rely on sphericity assumptions, making it particularly well suited for ERP data with multiple repeated measurements per participant (Tremblay and Newman, 2015; Barr et al., 2013).

For both analyses, the linear mixed-effects models included fixed effects of Referentiality (referential vs. bound-variable), Pronoun Type (masculine, feminine, gender-neutral), and their interaction. All fixed effects were coded using sum-to-zero contrasts, such that model coefficients reflect deviations from the grand mean and are appropriate for Type III hypothesis testing. Random intercepts and slopes for Referentiality and Pronoun Type by subject were specified, (1+Referentiality+Pronoun Type∣Subject), along with random intercepts for electrode site nested within subject to capture spatial variability, (1∣Subject:Electrode). Likelihood ratio tests confirmed that the nested electrode term significantly improved model fit for both gendered antecedents, χ^2^(1) = 44.67, *p* < 0.001, and non-gendered antecedents, χ^2^(1) = 20.95, *p* < 0.001. Including this term minimizes the multiple-comparisons burden and inflated familywise error rate associated with modeling scalp regions as fixed factors, increases power relative to models that treat Anteriority and Laterality as fixed effects, avoids the loss of information and underestimated standard errors that accompany averaging across electrodes, and more accurately reflects the nested structure of ERP data than a cross-classified model treating subject and electrode as independent random effects (Luck and Gaspelin, 2017). Models were estimated using restricted maximum likelihood (REML), and the Kenward-Roger approximation was applied to calculate denominator degrees of freedom and improve control of Type I error (Kenward and Roger, 1997). Fixed effects were tested using Type III Wald F tests. Estimated marginal means (EMMs), standard errors (SE), and 95% confidence intervals (CI) were obtained with the emmeans package (Lenth et al., 2024), and pairwise comparisons were Bonferroni-corrected for multiple testing. The full random effects structure was retained, in line with recommendations for confirmatory hypothesis testing in ERP research (Barr et al., 2013).

## 3 Results

### Non-gendered antecedents

This analysis examined whether pronouns carrying gender features would elicit mismatch-like responses when the antecedent itself lacked explicit gender information, particularly in bound-variable contexts where gender specification is not pragmatically licensed. Under this view, gender-marked pronouns—especially feminine forms—were expected to pattern differently from the gender-neutral reflexive when the antecedent was non-referential. The Type III ANOVA revealed a significant interaction between Referentiality and Pronoun Type, *F*_(2, 5584)_ = 24.99, *p* < 0.001, indicating that pronoun effects on P600 amplitude depended on whether the antecedent was referential or introduced in a bound-variable context (Table 2).

**Table 2:** Estimated marginal means by condition for non-gendered antecedents.

Referentiality	Pronoun type	Mean (μV)	SE	95% CI
Referential	Masculine-marked	0.53	0.13	[0.26, 0.80]
Referential	Feminine-marked	0.26	0.14	[−0.01, 0.53]
Referential	Gender-neutral	0.65	0.12	[0.41, 0.89]
Bound Variable	Masculine-marked	0.37	0.17	[0.04, 0.71]
Bound Variable	Feminine-marked	0.94	0.20	[0.53, 1.35]
Bound Variable	Gender-neutral	0.73	0.12	[0.49, 0.98]

Means are in microvolts.

Planned pairwise comparisons (Table 3) showed that this interaction was driven by bound-variable antecedents. In this context, feminine singular pronouns (“*someone…herself* ”) elicited significantly larger P600 amplitudes than masculine pronouns (“*someone…himself* ”), *t*_(47.9)_ = −3.21, *p* = 0.014, *d*_*z*_ = −0.52, 95% CI [−0.85, −0.18], consistent with a mismatch response to marked gender features in the absence of gender cues on the antecedent. By contrast, neither the difference between masculine and gender-neutral pronouns (“*someone…themselves*”), *t*_(43.1)_ = −2.15, *p* = 0.221, *d*_*z*_ = −0.35, 95% CI [−0.67, −0.02], nor the difference between feminine and gender-neutral pronouns, *t*_(42.6)_ = 1.18, *p* = 1.000, *d*_*z*_ = 0.19, 95% CI [−0.13, 0.51], survived Bonferroni correction. Singular “*they*” was therefore statistically indistinguishable from both the masculine and feminine forms individually; the only reliable contrast was between masculine and feminine reflexives. This pattern suggests that the P600 effect in this context reflects a difference between the two gender-marked poles rather than a straightforward violation response to marked gender features per se, with the gender-neutral reflexive occupying an intermediate position that does not reliably align with either gendered form.

**Table 3:** Planned pairwise contrasts and effect sizes for non-gendered antecedents.

Contrast	Estimate	SE	*t*(df)	*p* (adj)	*d*_*z*_	95% CI for *d*_*z*_
Referential congruent/masculine - referential incongruent/feminine	0.270	0.176	1.53(47.9)	0.795	0.248	[−0.07, 0.57]
Referential congruent/masculine - referential neutral	−0.119	0.175	−0.68(51.2)	1.000	−0.110	[−0.43, 0.21]
Referential neutral - referential incongruent/feminine	0.389	0.182	2.14(49.9)	0.223	0.347	[0.02, 0.67]
Bound variable congruent/masculine - bound variable incongruent/feminine	−0.567	0.176	−3.21(47.9)	0.014	−0.521	[−0.85, −0.18]
Bound variable congruent/masculine - bound variable neutral	−0.360	0.167	−2.15(43.1)	0.221	−0.350	[−0.67, −0.02]
Bound variable neutral - bound variable incongruent/feminine	0.206	0.174	1.18(42.6)	1.000	0.192	[−0.13, 0.51]

Reported statistics include estimated marginal mean differences, standard errors, *t*-values (with degrees of freedom), Bonferroni-adjusted *p*-values, Cohen's *d*_*z*_, and 95% confidence intervals for *d*_*z*_.

**Figure 1:**
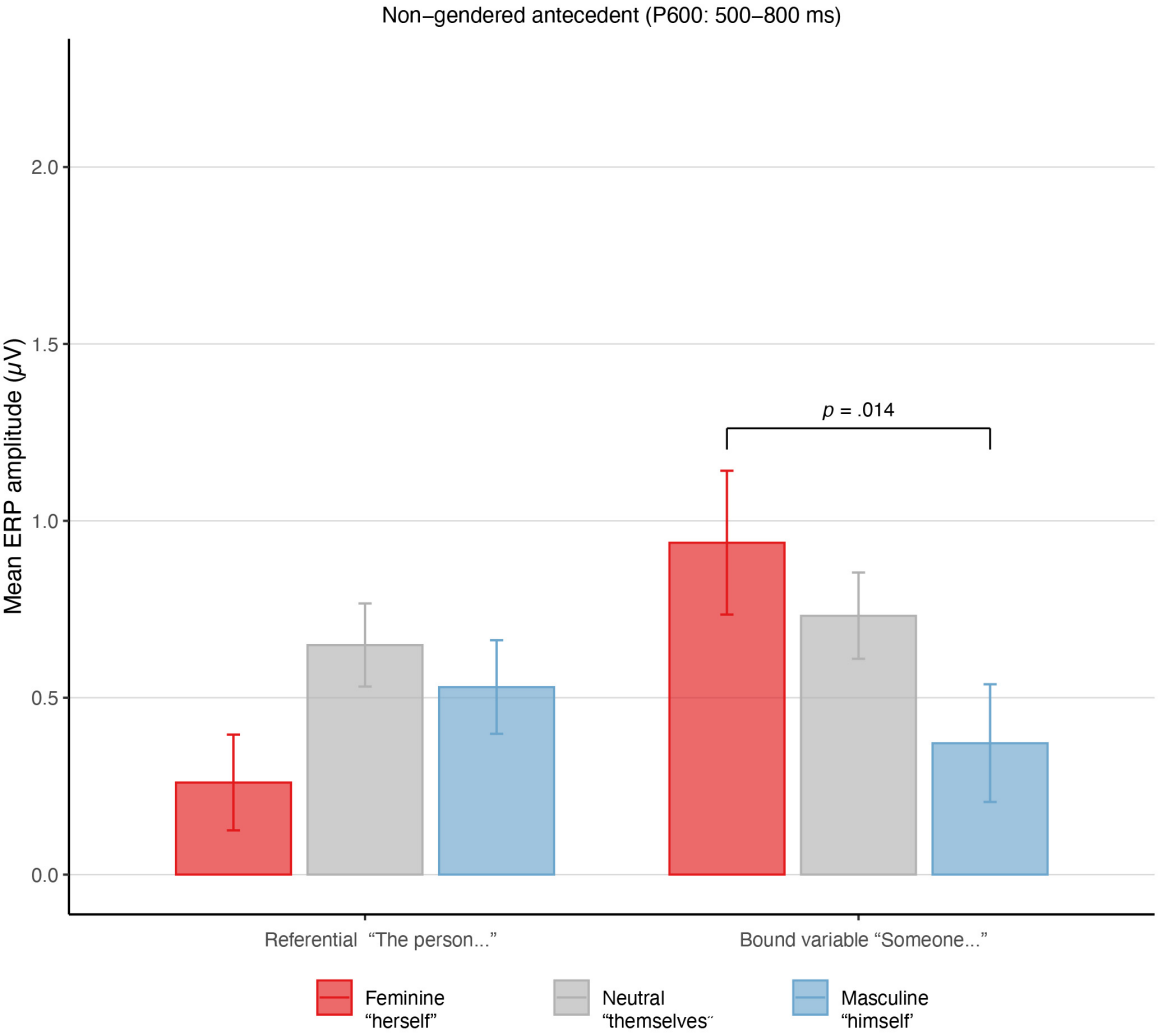
Mean ERP amplitudes (μV) in the 500–800 ms (P600) time window for reflexive pronouns co-indexed with non-gendered antecedents, shown separately for referential contexts (the person…) and bound-variable contexts (someone…). Error bars indicate ±1 standard error of the mean. Positive values reflect greater positivity in the P600 time window. In bound-variable contexts, feminine-marked reflexives elicited larger positivities than masculine-marked reflexives, whereas no comparable modulation was observed in referential contexts.

For referential antecedents (e.g., “*the person…*”), no contrasts were significant and all associated effect sizes were small (|*d*_*z*_| < 0.30), indicating an absence of violation-type responses in this context. Neither the main effect of Referentiality, *F*_(1, 37.32)_ = 2.15, *p* = 0.151, nor the main effect of Pronoun Type, *F*_(2, 36.33)_ = 1.10, *p* = 0.344, reached significance. Effect size estimates for these main effects were small ($\eta_{p}^{2}=0.05$ and .06, respectively), while the interaction accounted for a small but reliable proportion of variance ($\eta_{p}^{2}=0.009$). The model explained 1.2% of variance via fixed effects (Marginal *R*^2^ = 0.012) and 21.3% when random effects were included (Conditional *R*^2^ = 0.213; Figure 1).

### Gendered antecedents

This analysis tested the prediction that gender-incongruent pronouns would elicit robust mismatch responses relative to gender-congruent forms, with the nature of the response depending on antecedent type. The Type III ANOVA (Kenward-Roger approximation) revealed a strong interaction between Referentiality and Pronoun Type, *F*_(2, 5584)_ = 138.17, *p* < 0.001, indicating that pronoun effects on P600 amplitude differed as a function of whether the antecedent was referential or introduced in a bound-variable context (Table 4).

**Table 4:** Estimated marginal means by condition for gendered antecedents. Means are in microvolts.

Referentiality	Pronoun type	Mean (μV)	SE	95% CI
Referential	Gender-congruent	0.97	0.16	[0.66, 1.29]
Referential	Gender-incongruent	0.57	0.20	[0.17, 0.97]
Referential	Gender-neutral	0.79	0.12	[0.55, 1.03]
Bound Variable	Gender-congruent	0.26	0.16	[−0.07, 0.59]
Bound Variable	Gender-incongruent	1.75	0.19	[1.36, 2.14]
Bound Variable	Gender-neutral	0.56	0.14	[0.28, 0.83]

Planned pairwise comparisons (Table 5) showed that this interaction was driven by bound-variable antecedents: singular gender-incongruent pronouns (“*some woman…himself* ”) elicited substantially larger P600 amplitudes than gender-congruent pronouns (“*some woman…herself* ”), *t*_(43.8)_ = −7.03, *p* < 0.001, *d*_*z*_ = −1.14, 95% CI [−1.53, −0.74]. This contrast reflects a robust mismatch response characteristic of morphosyntactic repair processes. Critically, gender-incongruent pronouns also elicited significantly larger P600 amplitudes than gender-neutral pronouns (“*some woman…themselves*”) in bound-variable contexts, *t*_(42.1)_ = -5.70, *p* < 0.001, *d*_*z*_ = −0.925, 95% CI [−1.29, −0.55], indicating that singular “*they*” patterned away from the canonical mismatch response. By contrast, no reliable P600 differences were observed among pronoun types in referential contexts after correction, including comparisons between gender-incongruent and gender-neutral pronouns, consistent with the absence of a violation-type response for proper-name antecedents.

**Table 5:** Planned pairwise contrasts and effect sizes for gendered antecedents.

Contrast	Estimate	SE	*t*(df)	*p* (adj)	*d*_*z*_	95% CI for *d*_*z*_
Referential congruent - referential incongruent	0.404	0.212	1.91(43.8)	0.375	0.310	[−0.02, 0.63]
Referential congruent - referential neutral	0.184	0.157	1.17(46.8)	1.000	0.190	[−0.13, 0.51]
Referential neutral - referential incongruent	0.221	0.209	1.05(42.1)	1.000	0.171	[−0.15, 0.49]
Bound variable congruent - bound variable incongruent	−1.49	0.212	−7.03(43.8)	< 0.001	−1.14	[−1.53, −0.74]
Bound variable congruent - bound variable neutral	−0.294	0.157	−1.87(46.8)	0.403	−0.304	[−0.63, 0.02]
Bound variable neutral - bound variable incongruent	−1.19	0.209	−5.70(42.1)	< 0.001	−0.925	[−1.29, −0.55]

Reported statistics include estimated marginal mean differences, standard errors, *t*-values (with degrees of freedom), Bonferroni-adjusted *p*-values, Cohen's *d*_*z*_, and 95% confidence intervals for *d*_*z*_.

**Figure 2:**
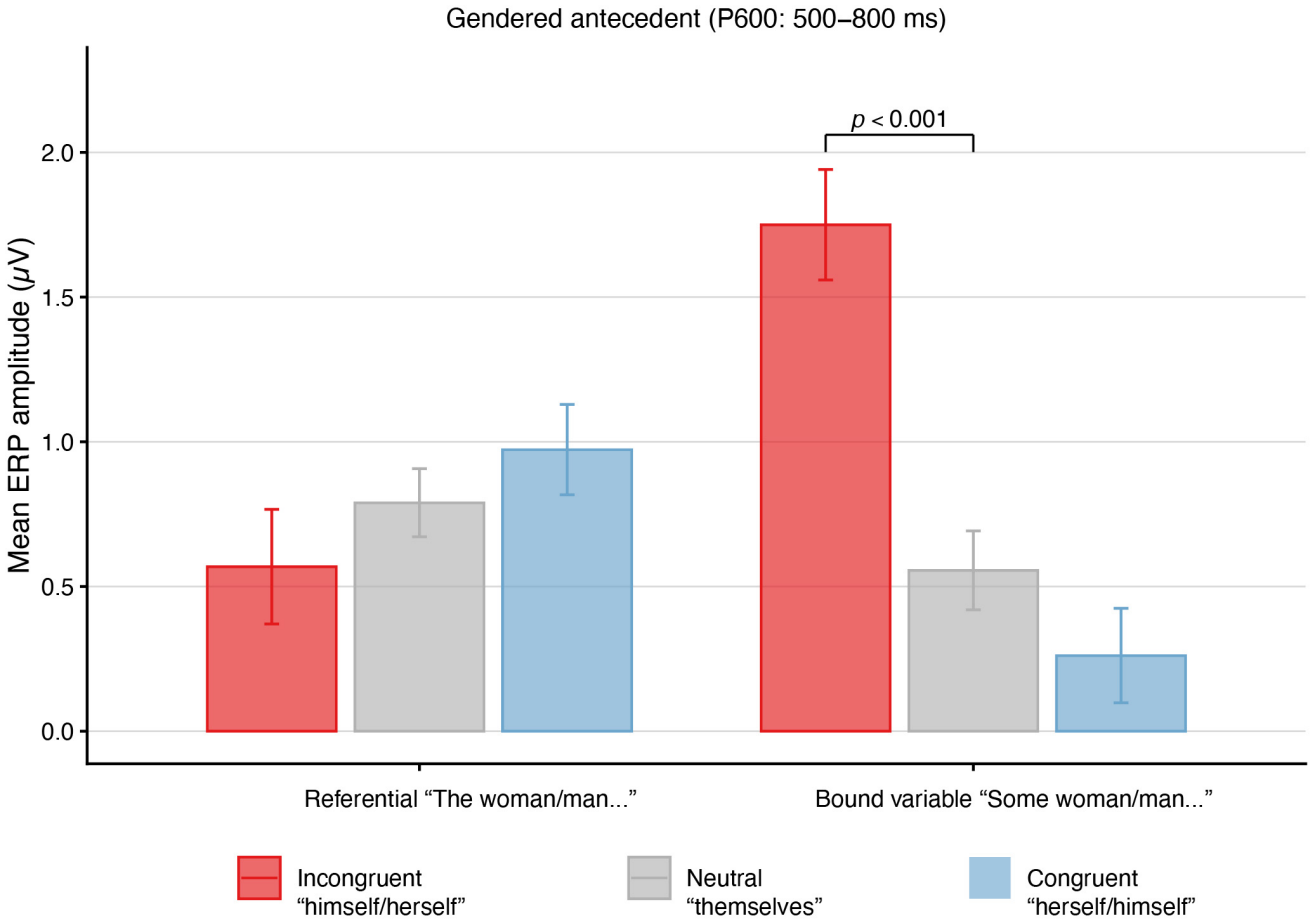
Mean ERP amplitudes (μV) in the 500-800 ms (P600) time window for reflexive pronouns co-indexed with gendered antecedents, shown separately for referential contexts (Mary…) and bound-variable contexts (Some woman…). Error bars indicate ±1 standard error of the mean. Positive values reflect greater positivity in the P600 time window. Gender-incongruent pronouns elicited enhanced P600 amplitudes in bound-variable contexts but not in referential contexts, consistent with a dissociation between structural and referential mismatch processing.

Although the main effect of Pronoun Type reached significance, *F*_(2, 36)_ = 3.68, *p* = 0.035, its interpretation is constrained by the strong interaction, and the main effect of Referentiality was not significant, *F*_(1, 37.98)_ = 0.40, *p* = 0.531. Effect size estimates indicated that the Referentiality × Pronoun Type interaction accounted for a modest proportion of variance ($\eta_{p}^{2}=0.05$), while Pronoun Type accounted for a moderate proportion ($\eta_{p}^{2}=0.17$). The model explained 4.5% of variance via fixed effects (Marginal *R*^2^ = 0.045) and 28.1% when random effects were included (Conditional *R*^2^ = 0.281; Figure 2).

Overall, for gendered antecedents, clear P600 mismatch responses emerged for gender-incongruent pronouns in bound-variable contexts, whereas singular “*they*” was statistically distinguishable from incongruent forms and did not elicit a comparable violation-type response.

### 3.1 Exploratory N400/Nref analysis

Although the P600 is considered the canonical ERP response to categorical morphosyntactic violations, negativities in the N400/Nref time window are more commonly associated with referential ambiguity or integration difficulty. While our primary analyses focused on the P600, the dissociation between referential and bound-variable contexts observed in prior ERP research motivated an exploratory analysis of mean amplitudes in the 300-500 ms window. This analysis was not part of the original analysis plan and is therefore interpreted descriptively. Because this analysis was intended to characterize the *type* of processing difficulty associated with pronoun-antecedent mismatches rather than to test equivalence between pronoun forms, contrasts were limited to theoretically diagnostic comparisons rather than the full set of planned comparisons used in the P600 analysis.

#### Non-gendered antecedents

For non-gendered antecedents, the same linear mixed-effects model structure was used, and inclusion of the nested electrode term again significantly improved model fit, χ^2^(1) = 40.31, *p* < 0.001. The Type III ANOVA revealed a significant Referentiality × Pronoun Type interaction,F_(2, 5014)_ = 19.98, *p* < 0.001 (Table 6), indicating that pronoun-related negativities differed across referential and bound-variable contexts.

**Table 6:** Estimated marginal means by condition for non-gendered antecedents in the 300–500 ms window. Means are in microvolts.

Referentiality	Pronoun type	Mean (μV)	SE	95% CI
Referential	Masculine-marked	0.77	0.17	[0.42, 1.12]
Referential	Feminine-marked	0.40	0.22	[−0.04, 0.84]
Referential	Gender-neutral	0.61	0.24	[0.12, 1.09]
Bound Variable	Masculine-marked	0.29	0.24	[−0.20, 0.77]
Bound Variable	Feminine-marked	0.80	0.24	[0.31, 1.28]
Bound Variable	Gender-neutral	0.35	0.19	[−0.04, 0.74]

Pairwise contrasts (Table 7) showed that the interaction was driven by bound-variable antecedents. In this context, masculine reflexives (“*someone…himself* ”; *M* = 0.29 μV, *SE* = 0.24) showed less positive—that is, more negative-going—amplitudes than feminine reflexives (“*someone…herself* ”; *M* = 0.80 μV, *SE* = 0.24), t_(44.5)_ = −2.09, *p* = 0.043, *d*_*z*_ = −0.34, 95% CI [−0.66, −0.01].

**Table 7:** Planned pairwise contrasts and effect sizes for gendered antecedents.

Contrast	Estimate	SE	*t*(df)	*p*	*d*_*z*_	95% CI for *d*_*z*_
Referential masculine - referential feminine	0.37	0.25	1.49(44.5)	0.143	0.24	[-0.08, 0.56]
Referential masculine - referential neutral	0.16	0.24	0.70(47.4)	0.491	0.11	[−0.21, 0.43]
Referential neutral - referential feminine	0.20	0.21	0.97(50.7)	0.339	0.16	[−0.16, 0.48]
Bound variable masculine - bound variable feminine	−0.51	0.25	−2.09(44.5)	0.043	−0.34	[−0.66, −0.01]
Bound variable masculine - bound variable neutral	−0.07	0.23	−0.29(41.5)	0.774	−0.05	[−0.37, 0.27]
Bound variable neutral - bound variable feminine	−0.45	0.20	−2.22(42.9)	0.032	−0.36	[−0.69, −0.03]

Reported statistics include estimated marginal mean differences, standard errors, *t*-values (with degrees of freedom), Bonferroni adjusted *p*-values, Cohen's *d*_*z*_, and 95% confidence intervals for *d*_*z*_.

The gender-neutral reflexive (“*someone…themselves*”; *M* = 0.35 μV, *SE* = 0.19) did not differ from masculine reflexives, *t*_(41.5)_ = −0.29, *p* = 0.774, *d*_*z*_ = −0.05, 95% CI [−0.37, 0.27], but showed nominally smaller amplitudes than feminine reflexives, *t*_(42.9)_ = −2.22, *p* = 0.032, *d*_*z*_ = −0.36, 95% CI [−0.69, −0.03]. This pattern suggests that the masculine/feminine difference in the N400/Nref window reflects primarily a contrast between the two gender-marked poles, with the gender-neutral reflexive occupying a numerically intermediate position. In referential contexts (e.g., “*the person…*”), no pairwise contrasts were significant and associated effect sizes were small: Masculine - Feminine, *t*_(44.5)_ = 1.49, *p* = 0.143, *d*_*z*_ = 0.24; Masculine–Neutral, *t*_(47.4)_ = 0.70, *p* = 0.491, *d*_*z*_ = 0.11; Feminine - Neutral, *t*_(50.7)_ = −0.97, *p* = 0.339, *d*_*z*_ = −0.16. As this analysis is exploratory, all contrasts are reported descriptively without correction for multiple comparisons.

Neither the main effect of Referentiality, *F*_(1, 37.25)_ = 0.38, *p* = 0.540, partial η^2^ = 0.01, nor the main effect of Pronoun Type, *F*_(2, 36.32)_ = 0.19, *p* = 0.826, partial η^2^ = 0.01, reached significance, although the interaction accounted for a small but reliable proportion of variance (partial η^2^ = 0.008). The model explained 0.7% of variance via fixed effects (Marginal *R*^2^ = 0.007) and 32.1% when random effects were included (Conditional *R*^2^ = 0.321 ).

#### Gendered antecedents

For gendered antecedents, analyses used the same linear mixed-effects model specification as in the P600 analysis (fixed effects of Referentiality, Pronoun Type, and their interaction; random slopes for Referentiality and Pronoun Type by subject; and nested electrode random intercepts). Including the nested electrode term significantly improved model fit, χ^2^(1) = 15.54, *p* < 0.001. The Type III ANOVA (Kenward-Roger approximation) revealed a significant Referentiality × Pronoun Type interaction, F_(2, 5014)_ = 43.18, *p* < 0.001 (Table 8), indicating that pronoun-related negativities differed across referential and bound-variable contexts.

**Table 8:** Estimated marginal means by condition for gendered antecedents in the 300–500 ms window. Means are in microvolts.

Referentiality	Pronoun type	Mean (μV)	SE	95% CI
Referential	Gender-congruent	0.86	0.23	[0.40, 1.32]
Referential	Gender-incongruent	−0.26	0.22	[−0.71, 0.19]
Referential	Gender-neutral	0.40	0.20	[−0.00, 0.80]
Bound variable	Gender-congruent	0.52	0.22	[0.09, 0.96]
Bound variable	Gender-incongruent	0.73	0.18	[0.37, 1.08]
Bound variable	Gender-neutral	0.28	0.14	[−0.00, 0.56]

Pairwise contrasts (Table 9) showed that this interaction was driven by referential antecedents , where the three reflexive types were ordered along a gradient in the N400/Nref direction. Gender-incongruent reflexives (“*Mary…himself* ”; *M* = −0.26 μV, *SE* = 0.22) showed significantly larger negativities than gender-congruent reflexives (“*Mary…herself* ”; *M* = 0.86 μV, *SE* = 0.23), *t*_(47.9)_ = 4.94, *p* < 0.001, *d*_*z*_ = 0.80, 95% CI [0.44, 1.15], consistent with increased referential or integration difficulty when a coreference interpretation is challenged. Critically, gender-congruent reflexives showed significantly less negative amplitudes than gender-neutral reflexives, *t*_(45.8)_ = 2.16, *p* = 0.036, *d*_*z*_ = 0.35 , 95% CI [0.02, 0.68], and significantly less negative amplitudes than gender-incongruent reflexives, *t*_(50.9)_ = 3.69, *p* < 0.001, *d*_*z*_ = 0.60, 95% CI [0.26, 0.94]. This three-step ordering—with gender-incongruent reflexives showing the greatest negativity, gender-neutral reflexives intermediate, and gender-congruent reflexives the least negative—is consistent with a gradient of predictability rather than a categorical distinction between matching and mismatching forms (see Section 4.1 for discussion).

**Table 9:** Pairwise contrasts and effect sizes for non-gendered antecedents in the 300–500 ms window.

Contrast	Estimate	SE	*t*(df)	*p*	*d*_*z*_	95% CI for *d*_*z*_
Referential congruent - referential incongruent	1.12	0.23	4.94(47.9)	< .001	0.80	[0.44, 1.15]
Referential congruent - referential neutral	0.46	0.21	2.16(45.8)	0.036	0.35	[0.02, 0.68]
Referential neutral - referential incongruent	0.65	0.18	3.69(50.9)	< .001	0.60	[0.26, 0.94]
Bound variable congruent - bound variable incongruent	−0.20	0.23	−0.90(47.9)	0.373	−0.15	[−0.46, 0.17]
Bound variable congruent - bound variable neutral	0.24	0.21	1.14(45.8)	0.261	0.19	[−0.14, 0.50]
Bound variable neutral - bound variable incongruent	−0.45	0.18	−2.52(50.9)	0.015	−0.41	[−0.74, −0.08]

This analysis was exploratory and not part of the original analysis plan; reported p-values are uncorrected and all results should be interpreted descriptively.

In bound-variable contexts, no contrasts involving congruent reflexives reached significance, indicating an absence of early negativities in contexts that instead elicited robust P600 responses. A nominally significant difference emerged between neutral and incongruent reflexives, *t*_(50.9)_ = -2.52, *p* = 0.015, *d*_*z*_ = −0.41, 95% CI [−0.74, −0.08], though given the exploratory nature of this analysis and the absence of a theoretically motivated account for this pattern in bound-variable contexts, this result should be interpreted cautiously. As this analysis is exploratory, all contrasts are reported descriptively without correction for multiple comparisons.

**Figure 3:**
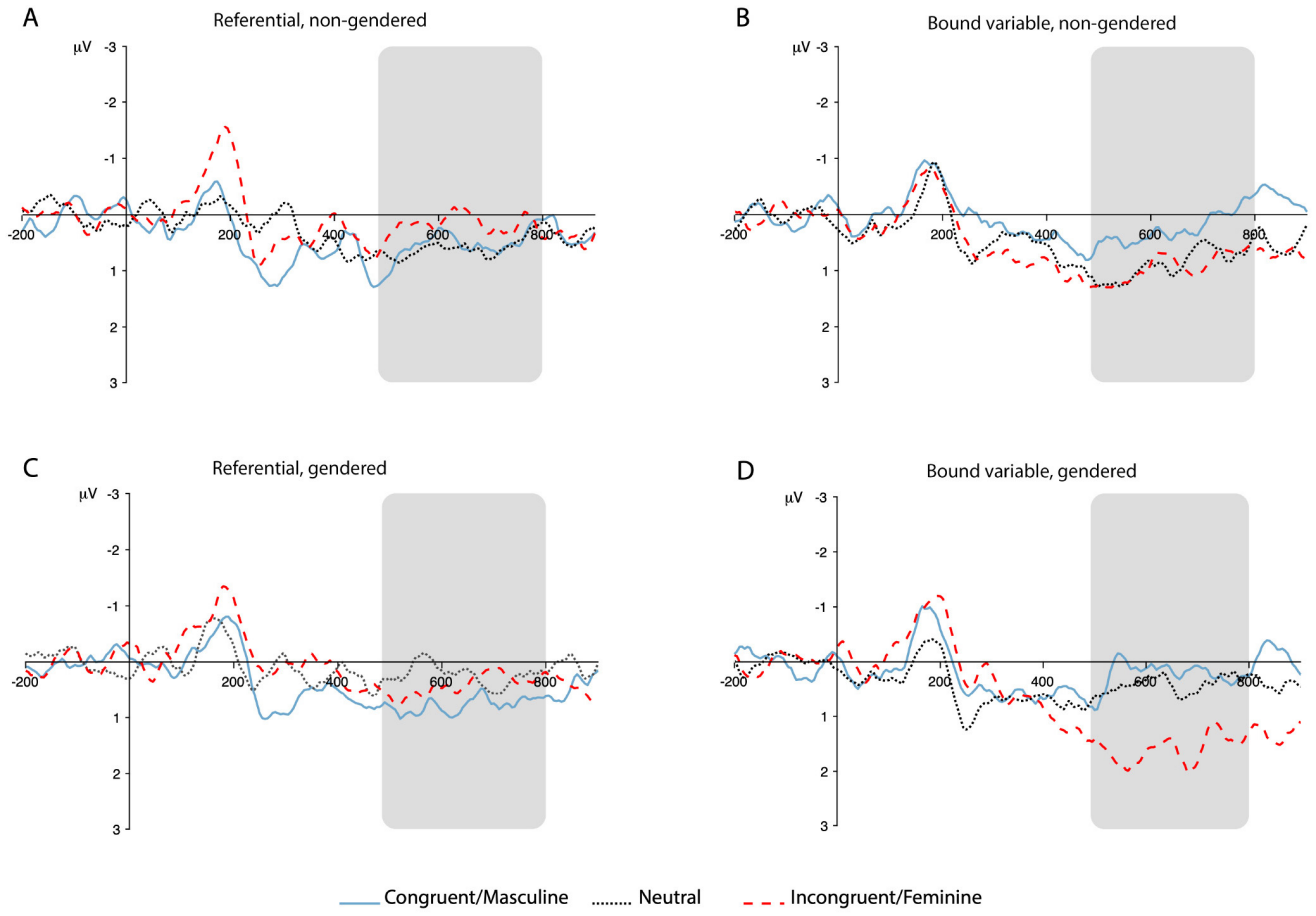
Grand-average ERP waveforms illustrating the P600 effect. Waveforms are averaged across a centro-parietal region of interest (TP7, CP3, CPz, CP4, TP8, P7, P3, Pz, P4, P8). Panels correspond to antecedent type: **(A)** referential, non-gendered; **(B)** bound-variable, non-gendered; **(C)** referential, gendered; **(D)** bound-variable, gendered. ERPs are time-locked to the onset of the critical word and plotted separately for the Congruent/Masculine, Neutral, and Incongruent/Feminine conditions. Negative voltage is plotted upward. The shaded region indicates the P600 analysis window (500–800 ms post-stimulus onset).

**Figure 4:**
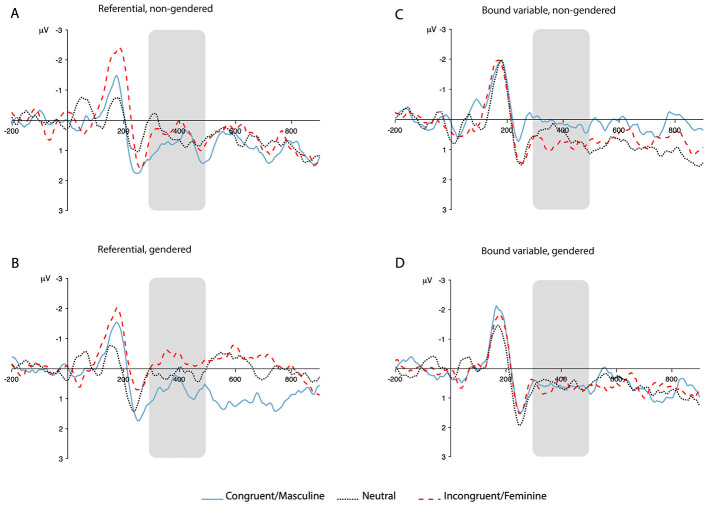
Grand-average ERP waveforms illustrating N400/Nref effects. Waveforms are averaged across a fronto-central region of interest (F7, F3, Fz, F4, F8, FT7, FC3, FCz, FC4, FT8). Panels correspond to antecedent type: **(A)** referential, non-gendered; **(B)** bound-variable, non-gendered; **(C)** referential, gendered; **(D)** bound-variable, gendered. ERPs are time-locked to the onset of the critical word and plotted separately for the Congruent/Masculine, Neutral, and Incongruent/Feminine conditions. Negative voltage is plotted upward. The shaded region indicates the N400/Nref analysis window (300–500 ms post-stimulus onset).

To illustrate the temporal evolution and scalp distribution of the observed effects, Figures 3, 4 present grand-average ERP waveforms for representative electrode clusters in the P600 and N400/Nref time windows. These waveforms are provided for visualization purposes and complement the statistical analyses reported above.

### 3.2 Summary of results

Across analyses, clear dissociations emerged between pronouns carrying gender features and gender-neutral forms and between referential and bound-variable contexts. For gendered antecedents, there were robust responses to gender-mismatching pronouns. In bound-variable contexts this response was a P600 effect, the classic response to agreement errors and other form-based anomalies, and singular “*themselves*” patterned closely with the gender-congruent pronouns. In referential contexts, gender mismatches resulted in N400/Nref effects, and the means for singular “*themselves*” were numerically intermediate between the congruent and incongruent gender-marked forms. We note, however, that since these N400/Nref analyses are exploratory and post-hoc, these findings should be interpreted descriptively rather than as confirmatory tests (see Section 4.1 for discussion).

For non-gendered antecedents, the pattern of effects across conditions was subtle and complex. In bound-variable contexts (e.g., “*someone*”), differences emerged in both time windows. One effect was predicted: feminine reflexives elicited larger P600 amplitudes than masculine reflexives, consistent with the marked gender form being treated as a mismatch in the absence of antecedent gender cues (*p* = 0.014; Table 3). The effect in the N400/Nref window was unexpected: masculine reflexives showed more negative-going amplitudes than feminine reflexives, which on a standard integration account would suggest that masculine forms were less predictable or more difficult to integrate—an unlikely interpretation given the markedness asymmetry discussed in Section 1.2. We cautiously interpret this pattern as potential component overlap, where an early-onset P600 for feminine forms artificially elevates feminine amplitudes in the N400/Nref window (see Section 4.2 for discussion).

In the non-gendered referential contexts, we predicted, and found, no mismatch or markedness effects. In the P600 window, there were no significant differences between any of the three reflexive forms, suggesting that all were equally compatible with this antecedent (masculine *p* = 0.795; neutral *p* = 1.000; feminine *p* = 0.223; see Table 3). Regarding the gender-neutral reflexive specifically, there was no significant difference between “*themselves*” and masculine reflexives in the N400/Nref window (*p* = 0.774), nor between “*themselves*” and feminine reflexives in the P600 window (*p* = 1.000), suggesting neither a markedness effect nor a processing penalty for singular “*themselves*” in these contexts. There was a small nominally significant difference between feminine and neutral reflexives in the N400/Nref window (*p* = 0.032, uncorrected), but given its small size and the exploratory nature of this analysis, we do not interpret this contrast further (see Table 7).

## 4 Discussion

The present study investigated how singular “*they*” is processed in real time when paired with gendered and non-gendered antecedents, with particular attention to how referential status shapes neural responses to pronoun-antecedent relations. The findings reveal a clear dissociation between bound-variable and referential contexts. In bound-variable contexts , gender-incongruent pronouns elicited robust P600 effects—a canonical signature of agreement violation—while singular “*they*” patterned closely with gender-congruent pronouns, showing no evidence of a comparable response. In referential contexts, the pattern was qualitatively different: gender-incongruent pronouns produced no P600 but instead elicited greater negativity in the N400/Nref time window relative to gender-congruent pronouns, and the N400/Nref response for singular “*they*” was intermediate between the gender-match and gender-mismatch conditions. As we discuss in Section 4.1, this gradient is consistent with a predictive coding account in which N400/Nref amplitude tracks the predictability of the incoming reflexive given the prior discourse context (Brothers et al., 2023; Nour Eddine et al., 2024): readers with high exposure to singular “*they*” appear to anticipate the gender-neutral reflexive, but less strongly or consistently than they anticipate the gender-congruent form.

Taken together, these findings indicate that in our study population, singular “*they*” is not treated as a grammatical error or mismatch. Even in contexts where the antecedent has highly stable gender features—such as quantified NPs like “*every woman*” or “*every man*”—which produce large P600 effects for mismatching reflexives, singular “*they*” elicits no comparable violation response. Moreover, our findings suggest that for this population any processing disruption associated with stereotypical gender expectations based on a proper name is quickly overcome, resulting in no mismatch response in the P600 time window. These findings counter the intuition that gender-neutral reflexives necessarily impose a processing cost when paired with gendered antecedents, at least for speakers with substantial exposure to singular “*they*”.

### 4.1 Antecedent type and ERP dissociations in pronoun processing

A central contribution of the present study is the dissociation observed in the neural responses to gender-mismatched pronouns across antecedent types. When mismatched reflexives followed proper name antecedents in referential contexts (e.g., “*Mary…himself* ”), they elicited frontal negativities in the N400/Nref time window with no reliable P600. When mismatched reflexives followed quantified antecedents in bound-variable contexts (e.g., “*every woman…himself* ”), they produced large P600 effects with no accompanying negativity. This dissociation is consistent with prior ERP research distinguishing referential integration difficulty from morphosyntactic reanalysis, but its precise source requires some care to interpret. In the present design, referential status and antecedent type are confounded: referential contexts used proper names, which carry strong but socially-mediated gender associations and introduce specific individuals whose identity is not shared with the reader, while bound-variable contexts used quantified NPs, which introduce structurally-specified gender variables rather than individual referents. The dissociation could therefore reflect the referential/bound-variable distinction per se, the difference between proper name antecedents and quantified NPs, or some combination of these factors. Future studies that independently manipulate referential status and antecedent type—for instance, by including definite descriptions in referential contexts or proper names in bound-variable contexts—would be needed to tease these possibilities apart.

The results in the bound-variable contexts are consistent with the extensive literature finding that gender or number mismatches on reflexive pronouns give rise to P600 effects when the antecedent is morphologically marked or the gender is definitional (Osterhout and Mobley, 1995; Osterhout et al., 1997; Canal et al., 2015). These effects are also typically present when the antecedent is a referential noun with a stereotypical gender, though they may be reduced in size or variable across participants (Canal et al., 2015; Osterhout et al., 1997). While these P600s are sometimes accompanied by anterior negativities, the negativities are often absent, particularly when the mismatch occurs in the middle of the sentence (Osterhout et al., 1997) or when the gender is definitional rather than stereotypical. This P600 is interpreted as evidence that a syntactic mismatch has been detected. The lack of a reliable and robust negativity suggests that this mismatch does not result in a search for a new antecedent, presumably because no other antecedent could bind the reflexive (see Xiang et al., 2009, and contrast with Nieuwland, 2014). A nominally significant difference between gender-incongruent and gender-neutral reflexives also emerged in the exploratory N400/Nref analysis for bound-variable contexts (*p* = 0.015, uncorrected; Table 9). This pattern is not readily interpreted in light of current theories of the N400 or the Nref. Taken at face value, it would suggest that gender-neutral forms were less predictable or harder to integrate than gender-incongruent forms in this context—a surprising conclusion given the linguistic distribution of these forms and the fact that a P600 was observed for the gender-incongruent form but not for the gender-neutral form. An alternative explanation is that this difference reflects an early manifestation of the P600 effect, measured here at fronto-central electrodes immediately preceding the centro-parietal window in which the dominant P600 was detected. Component overlap of this kind is common given the broad scalp distribution of P600 effects and the variability in their onset latency. Given that the effect is small, uncorrected, and part of an exploratory analysis, we flag it as a finding that warrants investigation in future work rather than drawing strong interpretive conclusions here.

The results in the referential context, where the antecedents are proper names, are more novel and more puzzling. We find no evidence of a P600 effect for the gender mismatches. We believe that this difference between the referential and bound-variable conditions is most plausibly attributed not to referentiality per se, but to the nature of the gender information provided by the antecedent. Quantified NPs such as “*every woman*”—or indeed any NP containing a gender-marked common noun such as “*the woman*” or “*the women*”—encode gender definitionally: the antecedent cannot refer to anything other than female individuals, and a masculine reflexive therefore constitutes a categorical structural incompatibility. Proper names, by contrast, carry probabilistic gender associations that are grounded in social convention rather than lexical specification. “*Sally*” is overwhelmingly but not exclusively female; “*John*” is overwhelmingly but not exclusively male. These associations can in principle be revised in light of new information without violating the grammar of the sentence. The difference between these antecedent types is thus not a difference in referential status, nor a difference in quantificational structure, but a difference in the rigidity of the gender commitment established by the noun itself.

The absence of a P600 in the proper name condition is all the more surprising because we used reflexive pronouns, which require coreference with the local antecedent and eliminate the possibility of introducing a new referent as a way of avoiding a mismatch interpretation. This sets our findings apart from those of (Nieuwland 2014), where anterior negativities in comprehension-focused tasks were attributed in part to participants' ability to pursue a non-coreference interpretation for non-reflexive pronouns. In the present study, no such strategy was available. We know of only two ERP studies of pronoun mismatch that use proper names to establish gender in a reflexive or strongly constrained context (Van Berkum et al., 2007; Qiu et al., 2012). Both find mismatch effects in at least some conditions. The absence of a comparable P600 in the present study may therefore reflect the more flexible gender-name mappings characteristic of our participant population—speakers with high familiarity with gender diversity for whom the association between a name like “*Sally*” and female gender may be less rigidly encoded as a grammatical constraint.

Nevertheless, the anterior negativity we observed for proper name gender mismatches demonstrates that our participants are sensitive to the probabilistic gender of names, even if this sensitivity does not generate a violation response. It is unlikely that this effect reflects the Nref in its classical sense, since Nref effects arise when referential ambiguity remains unresolved—for example, when a definite noun phrase or non-reflexive pronoun has multiple possible antecedents (Van Berkum et al., 2003). In contexts where the referent is strongly constrained but its features mismatch the pronoun, the Nref is typically absent and P600 effects occur instead (Van Berkum et al., 2007). Since reflexive pronouns must be bound within the clause, there is no referential ambiguity and an Nref would not be predicted.

This effect, however, bears a clear functional resemblance to the N400. N400 effects are observed when the critical word in one condition is less predictable than in another, and are interpreted as reflecting the degree to which lexical-semantic features can be pre-activated on the basis of prior context (Kutas and Federmeier, 2011; Nour Eddine et al., 2024). On this interpretation, the negativity around 400 ms reflects prediction of either the pronoun or its gender features on the basis of the name antecedent. When these predictions are falsified, our participants appear able to update their representation of the person under discussion—a process that is possible precisely because the name's gender association is probabilistic rather than definitional, and thus there is no grammatical violation requiring syntactic repair. This updating process produces no P600, consistent with studies that find N400 effects in the absence of P600 effects when the critical item is unexpected but not ungrammatical (Kuperberg et al., 2020). Crucially, the exploratory N400/Nref analysis reveals that this updating is gradient rather than categorical: the three reflexive types—gender-incongruent, gender-neutral, and gender-congruent—are ordered along a continuous predictability dimension in the N400/Nref window, with each step of the gradient statistically reliable (all *p* < 0.05, uncorrected; see Table 9). Readers most readily predict the gender-congruent form following a gendered proper name, predict the gender-neutral form to a lesser degree, and least readily predict the gender-incongruent form, consistent with a predictive coding account of the N400 (Nour Eddine et al., 2024). It is also worth noting that this negativity extends well beyond the classic N400 time window (see Figure 4), suggesting that the semantic updating process is sustained rather than fleeting.

Against this backdrop, singular “*they*” showed a markedly different processing profile from gender-incongruent pronouns across both antecedent types. In bound-variable contexts, singular “*themselves*” patterned away from the P600 mismatch responses observed for gender-incongruent pronouns, showing no reliable late positivity. In referential contexts, however, singular “*themselves*” showed an intermediate pattern, with N400/Nref amplitudes that were significantly more negative than those for gender-congruent reflexives but significantly less negative than those for gender-incongruent reflexives (*p* < 0.001 for both contrasts; see Table 9). This intermediate profile is consistent with the predictive framework outlined above: in referential contexts, singular “*themselves*” following a gendered proper name is less predictable than the gender-congruent form but more predictable than the gender-incongruent form, occupying an intermediate position in the predictability space established by the antecedent. In bound-variable contexts, by contrast, where gender is definitionally specified by the quantified antecedent, singular “*they*” escapes the categorical P600 response, suggesting that its underspecified features are simply incompatible with the violation detection mechanism rather than graded along a predictability dimension.

Before turning to the non-gendered antecedent results, we briefly consider an alternative interpretation of the null mismatch profile observed for “*themselves*”. Because “*themselves*” is ambiguous between singular and plural number, one might ask whether participants avoided treating it as a mismatch not because they parsed it as gender-underspecified, but because they reinterpreted the singular antecedent as a collective or group reference, thereby resolving an apparent number conflict pragmatically. We consider this account unlikely. It predicts that such reinterpretation should occur consistently across all singular antecedent types, including bare proper names such as “*Mary*” and “*John*”, which strongly resist collective readings in English. The implausibility of construing a named individual as a group referent makes this a high-cost interpretive move that would itself be expected to produce processing difficulty—difficulty that is not observed in the present data. Taken together, these observations are more consistent with the interpretation that “*themselves*” was processed as gender-underspecified, and thus compatible with a wide range of singular antecedents, than with an account in which number conflicts were routinely resolved through pragmatic reinterpretation.

### 4.2 Non-gendered antecedent mismatches

Non-gendered antecedents provide a complementary perspective on how gender features are introduced and evaluated during pronoun resolution. Because antecedents such as “*someone*” or “*the person*” do not lexically encode gender, they place fewer constraints on downstream pronouns, allowing gender features to be supplied—or left underspecified—at the level of the reflexive. Prior behavioral work suggests that singular “*they*” is generally well tolerated in such contexts, particularly in bound-variable constructions, whereas gender-marked pronouns may introduce additional interpretive commitments (Han and Moulton, 2022; Moulton et al., 2022; Foertsch and Gernsbacher, 1997).

Consistent with this view, pronouns carrying explicit gender features elicited differential neural responses with non-gendered antecedents. In particular, bound-variable pairings involving feminine reflexives (“*someone…herself* ”) elicited reliable P600 effects relative to masculine reflexives (“*someone…himself* ”), suggesting that in the absence of gender cues on the antecedent, the introduction of a marked gender feature at the reflexive can trigger reanalysis or repair processes indexed by late positivities. One explanation for this pattern is morphosyntactic markedness. In English, masculine-marked reflexives often function as the default option in contexts where gender is unspecified, whereas feminine-marked forms are more restricted and therefore impose stronger feature commitments. From this perspective, “*someone…himself* ” may be processed as an unmarked continuation that preserves gender underspecification, while “*someone…herself* ” forces the comprehender to introduce a specific gender feature not licensed by the antecedent. The resulting need to accommodate this feature is consistent with the emergence of a P600.

A complementary explanation comes from distributional frequency. Corpus data indicate that masculine reflexive pairings with non-gendered antecedents occur substantially more often than their feminine counterparts (Davies, 2008). Greater exposure to the masculine-marked sequence may increase its perceived naturalness and reduce processing demands, whereas the less frequent feminine-marked sequence may be treated as unexpected, again increasing the likelihood of reanalysis. Importantly, these accounts are not mutually exclusive: grammatical markedness and statistical frequency likely work together to shape expectations about how gender features are introduced in contexts where the antecedent itself is neutral.

Notably, the gender-neutral reflexive (“*someone…themselves*”) did not differ significantly from either gendered form in the P600 window, occupying a numerically intermediate position between masculine and feminine. This pattern is consistent with three possibilities. First, there could be variation in individual grammars such that some participants treat gender-neutral pronouns as grammatically marked relative to masculine pronouns (resulting in a P600) while others do not. Second, “*themselves*” may truly pattern consistently with “*herself* ” for all participants (suggesting it is grammatically marked), but power limitations prevent us from detecting this. Third, “*themselves*” may truly pattern with “*himself* ”, suggesting that both forms are unmarked. Disentangling these possibilities will require more data.

Our theoretical analysis has largely focused on the P600 responses for two reasons: P600 responses to gender-mismatch violations are well understood, and the P600 analyses were planned in advance. However, we should note that there was also an unexpected effect in the N400/Nref analysis. Specifically, we found a larger negativity to the masculine form than to the feminine form, which is unexpected on any theory. We suspect that this effect is attributable to component overlap—that the P600 for the feminine-marked reflexive is beginning to emerge in the 300–500 ms window, such that what appears to be a negativity to the masculine reflexive (reflecting an early integration cost) is actually a positivity to the feminine reflexive (reflecting morphosyntactic reanalysis). This interpretation is speculative, and alternative explanations cannot be ruled out. We flag it as a possibility that future studies examining the temporal dynamics of gender feature processing in non-gendered contexts should consider.

Taken together, the non-gendered antecedent results reinforce the broader conclusion that processing difficulty arises not simply from the presence of gender features per se, but from how and where those features are introduced in the pronoun-antecedent dependency. Singular “*they*”, by maintaining gender underspecification, appears to avoid some of the reanalysis processes engaged when marked gender features are added downstream, even in environments that otherwise permit flexible reference.

### 4.3 Population considerations and broader implications

An important consideration in interpreting the present findings is the linguistic experience of the participant sample. Many participants identified as non-binary or reported close personal connections to non-binary individuals, and thus are likely to have substantial everyday exposure to the referential use of singular “*they*”. This experience provides a meaningful context for understanding why singular “*they*” patterned away from the mismatch responses elicited by gender-specified pronouns, even in environments that strongly penalized incongruent uses of “*he*” and “*she*”. Rather than indicating a general absence of sensitivity to gender features, the results suggest that speakers with extensive exposure to singular “*they*” have integrated this form into their grammar in a way that accommodates co-indexation with gendered antecedents, whether those antecedents are proper names introducing individuals with probabilistic gender associations or quantified NPs specifying gender definitionally.

Crucially, this flexibility did not extend to gender-specified pronouns. Across analyses, mismatches involving “*he*” and “*she*” reliably elicited ERP signatures associated with referential difficulty or morphosyntactic reanalysis, indicating that sensitivity to gender features remained robust. The coexistence of these patterns suggests that experience with singular “*they*” does not weaken the grammatical system's ability to detect feature conflict more generally, but instead reflects a selective adaptation to a particular pronoun whose feature specification differs from traditional gendered forms.

A related design consideration concerns the form of the reflexive pronoun used in the gender-neutral condition. All stimuli employed “*themselves*” as the reflexive for singular “*they*” (e.g., “*John decided to treat themselves to sushi*”). However, for some speakers—particularly those who identify as nonbinary and those processing highly definite antecedents such as proper names—“*themself* ” may be the preferred or more natural singular reflexive form (Conrod et al., 2022). Given that over half of the present sample identified as nonbinary, it is possible that “*themselves*” carried some residual plurality for a subset of participants, introducing a mild processing cost relative to the more fully acceptable themself.

Importantly, however, if “*themselves*” were perceived as subtly plural-marked, this would be expected to work against the present findings rather than to inflate them. A plural-biased reflexive should elicit greater processing difficulty, making it harder, not easier, for the gender-neutral condition to pattern away from canonical mismatch responses. The fact that “*themselves*” consistently failed to elicit N400/Nref or P600 mismatch signatures across antecedent types suggests that the present conclusions are, if anything, conservative with respect to the acceptability of singular “*they*” for this population. Future research should directly compare “*themselves*” and “*themself* ” as reflexive forms, ideally with counterbalancing across participants, to determine whether reflexive form modulates online processing in speakers with high familiarity with singular “*they*”. Such studies should also retain trial-level data to allow post-hoc examination of order effects—for instance, whether participants who prefer “*themself* ” acclimate to “*themselves*” over the course of an experiment—and should include sufficient per-condition trials to support individual-level waveform analysis, which would allow researchers to examine whether any participants show ERP signatures consistent with treating “*themselves*” as a morphosyntactic error.

These observations highlight the importance of sociolinguistic experience in shaping real-time language processing. Rather than treating grammatical change as uniformly reflected across speaker populations, the present findings underscore that exposure and usage patterns can modulate how emerging forms are integrated at the neural level. Future work comparing populations with differing degrees of familiarity with singular “*they*” will be essential for determining how broadly these processing profiles generalize and for tracing how experience-driven adaptation interacts with more stable aspects of grammatical representation. Although the present study was designed to characterize processing in speakers with high familiarity, the findings raise a natural question about what would be expected for individuals with less exposure to singular they. Drawing on the existing behavioral and ERP literature, we can articulate three sets of predictions, ordered by their degree of theoretical certainty.

The most confident prediction is that low-exposure speakers would show reliable mismatch responses to themselves with gendered antecedents—responses that are absent in the present data. This prediction follows directly from behavioral work showing processing slowdowns for singular they with gendered antecedents in general population samples (Foertsch and Gernsbacher, 1997), and is consistent with the staged trajectory proposed by (Konnelly and Cowper 2020), in which speakers initially accept singular they only for referents of unknown or unspecified gender before extending this acceptance to clearly gendered individuals. Under this account, low-exposure speakers would be expected to remain at an earlier point in this trajectory, showing heightened sensitivity to the gender features of the antecedent and treating themselves as a mismatch particularly when paired with a strongly gendered referent.

A second, moderately confident prediction concerns the distribution of mismatch responses across antecedent types. Because bound-variable singular they has been in use for centuries and is broadly accepted even among speakers who resist its referential use (Bjorkman, 2017; Han and Moulton, 2022; Moulton et al., 2022), low-exposure speakers would be expected to show an asymmetry between contexts: mismatch responses should be more pronounced in referential contexts—particularly with proper name antecedents, which introduce highly specific discourse referents with stable gender associations — than in bound-variable contexts, where quantified antecedents impose fewer constraints on downstream gender specification. This asymmetry mirrors the referential/bound-variable dissociation observed in the present data for gender-incongruent pronouns, and suggests that even less experienced speakers may retain some sensitivity to the structural licensing conditions that differentiate these two uses.

A third, more speculative prediction concerns the type of mismatch response that low-exposure speakers would exhibit. Within the ERP literature, whether a mismatch elicits a P600 or an N400/Nref depends in part on whether comprehenders treat the pronoun-antecedent relation as a categorical agreement violation or as a failure of referential integration (Nieuwland, 2014). Speakers who have little experience with singular “*they”* and who are therefore more likely to persist in treating it as an agreement violation may show P600-dominant mismatch profiles, whereas speakers with intermediate exposure—sufficient to prevent a strict violation interpretation but insufficient to fully integrate singular they into their pronoun resolution system—may instead show N400/Nref-dominant profiles reflecting referential difficulty. If this prediction is borne out, it would suggest that the shift from mismatch to null processing observed in the present high-exposure sample represents the endpoint of a gradient continuum, rather than a categorical difference between speakers who have and have not encountered singular “*they”*. Testing these predictions directly, through studies that sample participants across the full range of familiarity with singular “*they”*, would provide a powerful test of the experience-driven adaptation account and would substantially clarify the mechanisms through which sociolinguistic change is reflected in real-time language processing.

### 4.4 General conclusions

Together, these findings reveal that the neural response to pronoun-antecedent mismatches depends critically on the nature of the gender information the antecedent provides. When gender-specified pronouns conflicted with the definitional gender features of quantified antecedents, we observed robust P600 responses, a pattern characteristic of grammatical errors and morphosyntactic mismatches. When gender-specified pronouns followed proper name antecedents with probabilistic gender associations, we observed N400 effects, suggesting that the mismatching form was unexpected but not treated as unacceptable. For singular “*they*”, there was no evidence of a violation response in either context. In referential contexts, “*they*” showed an intermediate N400 profile—more negative than the gender-congruent form but reliably less negative than the gender-incongruent form—consistent with a form that is somewhat unpredicted but readily accommodated. Thus singular “*they*” exhibited a distinct processing profile across antecedent types, consistent with its underspecified gender representation and with the flexible grammatical integration that characterizes speakers with extensive exposure to its use. By documenting how the type of gender information encoded by the antecedent shapes both the nature of mismatch responses and the processing of singular “*they*”, the present study demonstrates how ERPs can illuminate the interaction of grammar, discourse, and sociolinguistic experience as the English pronoun system continues to evolve.

## Data Availability

The datasets presented in this study can be found in online repositories. The names of the repository/repositories and accession number(s) can be found below: The datasets analysed for this study are publicly available on OSF at https://osf.io/2vjyp/.

## Footnotes

1 Although most prior studies have used visual presentation, (Hagoort and Brown 2000) found that when sentences were presented in the auditory modality, syntactic violations elicited a P600 with the same onset as was observed for the reading of these sentences.
